# Efficacy and Safety of *Melaleuca alternifolia* (Tea Tree) Oil for Acne—A Systematic Review and Meta‐Analysis

**DOI:** 10.1002/ptr.70344

**Published:** 2026-05-07

**Authors:** Yixin Ye, Jun Jie Lim, Zongxun Huang, Fook Tim Chew

**Affiliations:** ^1^ School of Public Health and Management Guangxi University of Chinese Medicine Nanning China; ^2^ Department of Biological Sciences, Faculty of Science National University of Singapore Singapore Singapore

**Keywords:** acne vulgaris, efficacy, meta‐analysis, safety, tea tree oil (TTO)

## Abstract

Acne vulgaris is a common chronic inflammatory skin disorder that begins in adolescents and often persists into adulthood, with substantial psychosocial impact. Tea tree oil (TTO) has been explored as a topical treatment for acne, but clinical evidence for its efficacy and safety remain limited and inconsistent. This systematic review and meta‐analysis synthesizes available clinical data to evaluate TTO's therapeutic effects and safety profile in acne management. PubMed, Embase, and Web of Science were systematically searched for clinical studies published up to August 2025 that evaluated TTO for acne treatment. Study selection, data extraction, and risk‐of‐bias assessment were independently conducted by trained reviewers. Pooled estimates were calculated using fixed‐ or random‐effects models according to heterogeneity, quantified using the *I* ^2^ statistic. Seven studies comprising 445 patients were included. The use of TTO was associated with a reduction in acne severity compared with control groups (pooled odds ratio [pOR] = 0.74, 95% confidence intervals [CI]: 0.63–0.88). Adverse events were predominantly local and mild. An exploratory pooled analysis indicated a lower incidence of mild itching with TTO compared to the corresponding control treatments in the included studies (pOR = 0.09, 95% CI: 0.03–0.23), with similar trends observed for dryness, burning sensation, erythema, and scaling. Publication bias was not evident based on Egger's test and the trim‐and‐fill analysis, and overall risk of bias was assessed as low. Current evidence suggests that TTO is associated with a modest reduction in acne severity and generally acceptable short‐term tolerability. Larger, high‐quality trials with standardized formulations and long‐term follow‐up are needed to define its clinical role.

## 1 Introduction

Acne vulgaris is the most prevalent chronic inflammatory dermatosis, affecting up to 96% of adolescents, with symptoms often persisting into early adulthood or beyond (Heng and Chew 2020; Layton et al. 2022). In 2021, the global age‐standardized prevalence was 25% higher in young women (10,911.8 per 100,000) than in young men (8727.8 per 100,000) (Zhu et al. 2025). Acne imposes a significant disease burden worldwide, adversely affecting both physical appearance and quality of life, particularly among young adults (Chen et al. 2022; Naldi 2022). Beyond its cosmetic impact, the condition can cause psychological distress and is frequently associated with erythema, hyperpigmentation, and persistent scarring (Dale et al. 2024). The pathogenesis of acne is multifactorial, involving androgen‐driven sebum overproduction, follicular hyperkeratinization and obstruction, colonization by *Cutibacterium acnes* (formerly *Propionibacterium acnes*), and perifollicular inflammation (Barbieri 2020; Xu et al. 2025). Current treatment selection is guided by acne severity, typically assessed using standardized grading scales such as the Investigator's Global Assessment (IGA). For mild‐to‐moderate acne, topical therapies (e.g., retinoids, benzoyl peroxide, and antibiotics) used in combination are recommended. In contrast, severe acne generally requires systemic treatment, including oral antibiotics or isotretinoin, with topical agents serving an adjunctive role (Barbieri 2020; Reynolds et al. 2024).

Tea tree oil (TTO), an essential oil derived from the leaves of *Melaleuca alternifolia*, is widely used for various dermatological conditions, including acne. Its primary active compound, terpinen‐4‐ol, constitutes 35%–48% of TTO and is considered responsible for its therapeutic effects (Kairey et al. 2023; Nascimento et al. 2023). In recent years, TTO has attracted growing interest due to its reported broad‐spectrum antimicrobial and anti‐inflammatory properties, and its potential role as a topical alternative or adjunct to conventional acne therapies. It has also gained attention among consumers seeking non‐pharmacological options for acne management (Carson et al. 2006; Yadav et al. 2016). TTO has demonstrated in vitro activity against *C. acnes* and has been suggested to influence sebum production and local inflammatory responses (Nascimento et al. 2023). Its proposed antimicrobial mechanisms include disruption of bacterial cell membranes, intereference with cellular respiration, and inhibition of microbial proliferation (Iacovelli et al. 2023). However, the extent to which these mechanisms translate into clinically relevant effects remains uncertain. Despite this, TTO has been considered as a potential complementary or alternative option for acne, particularly in the context of increasing concerns about antibiotic resistance. Historically, TTO has been used in traditional medicine for treating wounds, burns, insect bites, and respiratory conditions (Chin and Cordell 2013; Kairey et al. 2023), and is now incorporated into a range of cosmetic and pharmaceutical products, including shampoos, soaps, and mouthwashes (Kunicka‐Styczyńska et al. 2009, 2011; Wiatrak et al. 2021).

Despite encouraging preclinical findings, the clinical efficacy and safety profile of TTO for acne remain insufficiently defined. While TTO is generally considered safe for topical use at low concentrations (Bekhof et al. 2023; Kairey et al. 2023), this perception is largely based on evidence of heterogeneous quality and limited standardized safety evaluation. Existing systematic and narrative reviews have summarized TTO's bioactive properties and dermatological applications, including acne management (Kairey et al. 2023; Nascimento et al. 2023). However, their conclusions are understandably constrained by inconsistent efficacy estimates, incomplete reporting of adverse events, reliance on non‐systematic study selection, and the absence of formal risk‐of‐bias assessment. Moreover, several recent clinical trials have not been comprehensively integrated into these syntheses.

To address these limitations, this study undertakes a systematic review and meta‐analysis using predefined eligibility criteria, structured quality appraisal, and quantitative synthesis to deliver a more rigorous and up‐to‐date evaluation of TTO's efficacy and safety in acne treatment. The primary objectives are to compare the therapeutic efficacy of TTO with placebo and to quantify the incidence of treatment‐related adverse events. Secondary objectives include characterizing the type and frequency of reported side effects and examining their association with clinical outcomes. By systematically synthesizing available clinical evidence, this updated study aims to clarify the therapeutic role of TTO within contemporary acne management.

## 2 Methods

### 2.1 Search Strategy

The protocol for this systematic review and meta‐analysis was prospectively registered with the International Prospective Register of Systematic Reviews (PROSPERO) under registration number CRD420251127442. This study was conducted in accordance with the Preferred Reporting Items for Systematic Reviews and Meta‐Analyses (PRISMA) guidelines (Page et al. 2021). The completed PRISMA 2020 checklist is available as Data S1.

A comprehensive literature search was conducted in the PubMed, Embase, Web of Science, Cochrane Library, and Ovid databases for clinical studies on the use of TTO in acne treatment, published up to August 8, 2025. To ensure a broad search scope, terms including “acne,” “acne vulgaris,” “tea tree,” *Melaleuca alternifolia* oil, and *Aetheroleum Melaleucae alternifoliae* were utilized, combining both subject headings and free‐text terms. The detailed search strategy for PubMed is provided in Table 1.

**TABLE 1:** Retrieval strategy in PubMed.

Steps	Search terms
#1	(“Melaleuca alternifolia”[Mesh]) OR (“Tea Tree Oil”[Title/Abstract]) OR (“Melaleuca Oil”[Title/Abstract]) OR (“Tea Tree Extract”[Title/Abstract])
#2	(“Acne”[Mesh]) OR (“Acne Vulgaris”[Title/Abstract]) OR (“Pimples”[Title/Abstract]) OR (“Acne Lesions”[Title/Abstract]) OR (“Acne Severity”[Title/Abstract])
#3	(“Efficacy”[Title/Abstract]) OR (“Effectiveness”[Title/Abstract]) OR (“Therapeutic”[Title/Abstract]) OR (“Treatment Outcome”[Title/Abstract]) OR (“Clinical Outcome”[Title/Abstract])
#4	(“Safety”[Title/Abstract]) OR (“Adverse Effects”[Title/Abstract]) OR (“Side Effects”[Title/Abstract]) OR (“Toxicity”[Title/Abstract])
#5	#1 AND #2 AND (#3 OR #4)

### 2.2 Study Selection

Two independent reviewers screened the titles and abstracts to determine eligibility. If either reviewer considered a title or abstract to be potentially relevant, the full text of the article was retrieved and assessed. The reviewers then critically evaluated the methodology and outcomes of the included studies. Article extraction was performed independently, and any disagreements were resolved through discussion with a third reviewer to reach a consensus.

The inclusion criteria were as follows: (a) human clinical trials; (b) studies evaluating TTO for the treatment of acne; (c) participants diagnosed with acne vulgaris, with severity ranging from mild to severe; (d) studies reporting the number of facial acne lesions; and (e) provision of original data with sufficient information to calculate outcomes. The exclusion criteria were: (a) non‐original articles (e.g., reviews, case reports, or commentaries); (b) nonhuman studies (e.g., animal or in vitro studies); (c) duplicate publications; (d) articles with insufficient data; or (e) articles not published in English.

### 2.3 Data Extraction and Quality Assessment

Data extracted from each study included author, publication year, country, study design, comparison groups, details of the TTO intervention, primary outcomes (e.g., reduction in inflammatory acne lesions and change in Acne Severity Index [ASI]), and reported adverse events (e.g., skin dryness, itching, stinging, burning, and erythema). Data extraction discrepancies were resolved by consensus. Two reviewers independently assessed risk of bias using the revised Cochrane Risk of Bias tool for randomized controlled trials (Higgins et al. 2011; Sterne et al. 2019). This tool evaluates bias across six domains: random sequence generation, allocation concealment, blinding of participants and personnel, incomplete outcome data, selective reporting, and other potential biases. Each domain was rated as low, high, or unclear risk. Disagreements were resolved through discussion with a third reviewer.

### 2.4 Statistical Analysis

Between‐study heterogeneity was assessed using the chi‐squared test and quantified with the *I* ^2^ statistic. A fixed‐effects model was applied if *I* ^2^ ≤ 50% and the corresponding *p* > 0.10; otherwise, a random‐effects model was applied to estimate the pooled odds ratios (pORs) with 95% confidence intervals (CIs).

#### 2.4.1 Definition of Comparison Groups

In trials where TTO was the primary intervention (Enshaieh et al. 2007; Malhi et al. 2017; Najafi‐Taher et al. 2022), comparators included placebo or standard topical agents (e.g., 0.1% adapalene gel). In contrast, Kwon et al. (2014) employed a split‐face design in which TTO functioned as the control against another botanical intervention (*Lactobacillus*‐fermented *Chamaecyparis obtusa* extract, LFCO). Accordingly, efficacy data from Kwon et al. (2014) were excluded from the meta‐analysis of TTO efficacy but retained for safety analyses, as the TTO arm provided relevant tolerability data.

For the primary efficacy outcome, we established a post hoc dichotomous outcome termed “clinically significant improvement,” defined as either a ≥ 50% reduction in total lesion count or attainment of “clear”/“almost clear” skin. This was derived from the two trials where TTO was the primary intervention. For Malhi et al. (2017), we calculated the proportion of participants achieving the ≥ 50% reduction threshold (more stringent than the original 40% efficacy criterion). For Najafi‐Taher et al. (2022), we used the reported rate of treatment success, which corresponded to our definition of clinical improvement. pORs were then calculated by comparing the TTO groups to their respective control groups within these two studies.

Subgroup analyses (e.g., by TTO concentration or participant characteristics) were considered but not formally tested. Sensitivity analyses assessed robustness to model choice (fixed vs. random effects including DerSimonian–Laird and restricted maximum likelihood (REML) methods) and exclusion of studies at high bias of risk. The primary outcomes, including reductions in inflammatory acne lesions, changes in ASI, and safety outcomes (mild itching, dryness, erythema), were analyzed separately. Prior to pooling, outcomes were harmonized through standardization of measurement units and ensuring methodological consistency across studies to allow for accurate comparisons. Publication bias was assessed using Egger's test. All statistical analyses were performed using Stata version 14.0 (Stata Corp, College Station, TX).

## 3 Results

### 3.1 Study Selection and Characteristics

**FIGURE 1:**
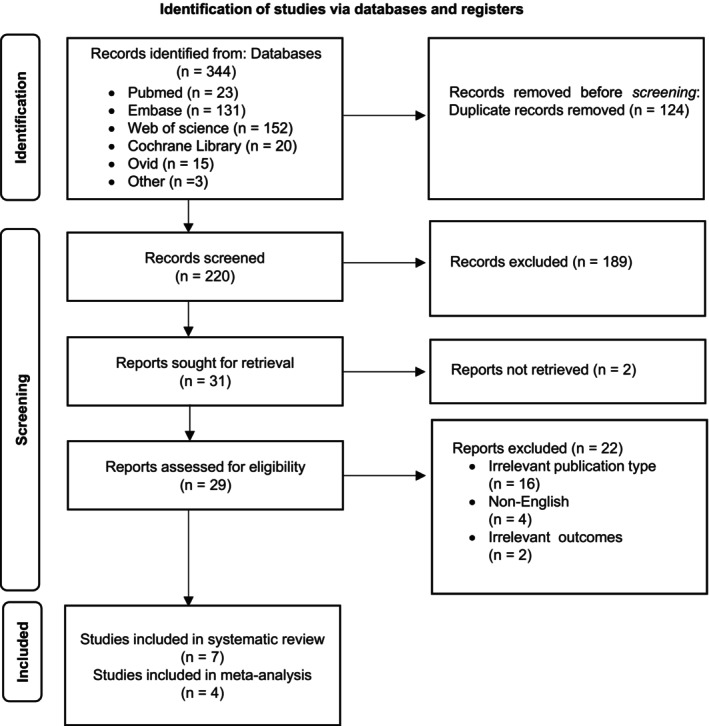
PRISMA flow diagram illustrating the study selection process for the systematic review and meta‐analysis of Tea Tree Oil on acne vulgaris.

A systematic search of electronic databases yielded 344 records: PubMed (*n* = 23), Embase (*n* = 131), Web of Science (*n* = 152), the Cochrane Library (*n* = 20), and Ovid (*n* = 15). Three additional records were identified from other sources. After removing 120 duplicates, two independent reviewers screened the titles and abstracts of 220 unique records. Of these, 189 were excluded for being irrelevant to the topic or for being review articles. The remaining 31 full‐text articles were assessed for eligibility, and 22 were excluded for the following reasons: non‐English language, absence of relevant data, or not being a research article. Consequently, seven studies were included in the qualitative synthesis. However, only four of these studies met the criteria for quantitative synthesis (meta‐analysis), as detailed in Table 2. The three studies excluded from the meta‐analysis (see Table S1) were omitted due to insufficient data for calculating effect sizes, a lack of homogeneity in study designs, or differences in outcome measures that precluded a valid comparison. This rigorous selection process ensured the inclusion of high‐quality and relevant studies. The study selection process is illustrated in Figure 1.

**TABLE 2:** General information included in the study.

Included studies	Country	Sample size (treatment/control)	Intervention group	Treatment plan	Main findings
Kwon et al. (2014)	Korea	34/34	5% TTO	Twice a day, with a cycle of 8 weeks.	After 8 weeks, the inflammatory acne lesions on the TTO side decreased by 38.2%. The subjective satisfaction of patients is also high. In addition to this clinical efficacy, 4 cases (12.5%) reported mild dryness, and 6 cases (18.8%) reported mild erythema and peeling.
Bassett et al. (1990)	Australia	124/61	5% TTO	The intervention period is 3 months.	44% (27/61) of patients treated with TTO reported adverse reactions, mainly associated with dry skin, itching, stinging, burning, and redness.
Enshaieh et al. (2007)	Iran	60/30	5% TTO	Applied twice a day to the acne area for 20 min, then rinsed with tap water, and continued treatment for 45 days.	There were significant differences between TTO gel and placebo in the improvement of total acne focus count (TLC) and ASI. In terms of TLC and ASI, the effectiveness of TTO gel is 3.55 times and 5.75 times higher than that of placebo respectively. In the 5% TTO treatment group, 3 out of 30 patients (10%) reported the slightest itching, 1 patient (3.33%) reported a slight burning sensation during medication use, and another patient (3.33%) reported the slightest scab sensation.
Najafi‐Taher et al. (2022)	Iran	100/47	TTO nanoemulsion containing adapalene gel	Used once a night for 12 weeks.	According to the change of acne severity index, 71.69% of patients in the TTO NE + ADA gel group succeeded in the treatment, while the proportion in the control group was 6.38%. Furthermore, no increase in the severity level of acne was observed in any group of patients.
Malhi et al. (2017)	Australia	14/0	TTO gel (200 mg/g)	Applied to the affected area on the face twice a day and maintain the product for at least 6 h for 12 weeks.	The average percentage decrease in total lesion count from baseline was 25% at 4 weeks, 37% at 8 weeks, and 54% at 12 weeks. The total lesion count for each visit varied significantly (*p* < 0.0001) due to repeated measurements. The product showed clinical efficacy in 11 participants (79%). No serious adverse events occurred.
Mazzarello et al. (2018)	Italy	60/20	20% propolis extract, 3% TTO, and 10% *Aloe vera* leaf juice (PTAC)	Used twice a day, in the morning and evening, for 30 days.	After 15 and 30 days of PTAC application, the cheekbone and scar lesions showed a significant reduction in erythema.
Infante et al. (2023)	Brazil	53/14	2% *Melaleuca alternifolia* (tea tree)	Used daily for 90 days.	The nanoemulsion of *M. alternifolia* demonstrated the most promising outcomes in reducing comedone areas, especially in the infundibular region.

### 3.2 Quality Assessment

The seven included studies consisted of three randomized, double‐blind, placebo‐controlled trials; two randomized, double‐blind, comparative studies; one single‐blind, randomized clinical trial; and one non‐controlled, open‐label study. The quality of these studies was evaluated using the Cochrane Risk of Bias Tool. Most studies demonstrated a low risk of bias in random sequence generation and blinding of outcome assessment, indicating generally robust methodologies. However, variability was observed in several domains, including allocation concealment, blinding of participants and personnel, incomplete outcome data, and selective reporting. Specifically, the studies by Bassett et al. (1990) and Enshaieh et al. (2007) exhibited a low risk of bias across domains. In contrast, the studies by Malhi et al. (2017) and Mazzarello et al. (2018) showed a higher risk associated with allocation concealment and blinding. Furthermore, studies by Infante et al. (2023) and Najafi‐Taher et al. (2022) were associated with an increased risk due to incomplete outcome data, while the study by Kwon et al. (2014) showed a higher associated risk in the blinding of participants and personnel. Detailed risk‐of‐bias assessments are provided in Figure S1.

### 3.3 Efficacy Results

**FIGURE 2:**
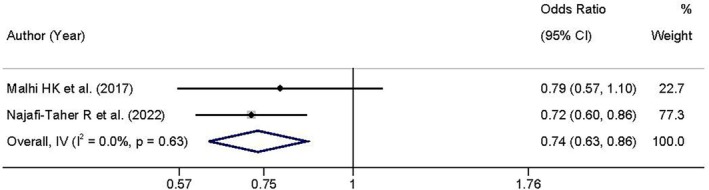
Forestplot of the efficacy of tea tree oil (TTO) on acne treatment.

The primary efficacy outcome was the proportion of participants achieving clinically significant improvement, which we defined as a ≥ 50% reduction in total lesion count or an equivalent level of clearance as reported by the original study (refer to Section 2). As this outcome was dichotomous, we pooled ORs rather than continuous measures. pORs quantify the relative odds of achieving this improvement with TTO‐based treatment compared to control, which was either 0.1% adapalene gel or, for the single‐arm study, a theoretical benchmark based on our ≥ 50% reduction criterion. A fixed‐effect meta‐analysis yielded a summary pOR of 0.74 (95% CI: 0.63–0.86; *I* ^2^ = 0.0%), indicating homogeneous evidence (*p* = 0.60) of an association between TTO treatment and reduced acne severity (Figure 2). The funnel plot (Figure S2) shows asymmetry, suggesting potential publication bias.

### 3.4 Safety Results

**FIGURE 3:**
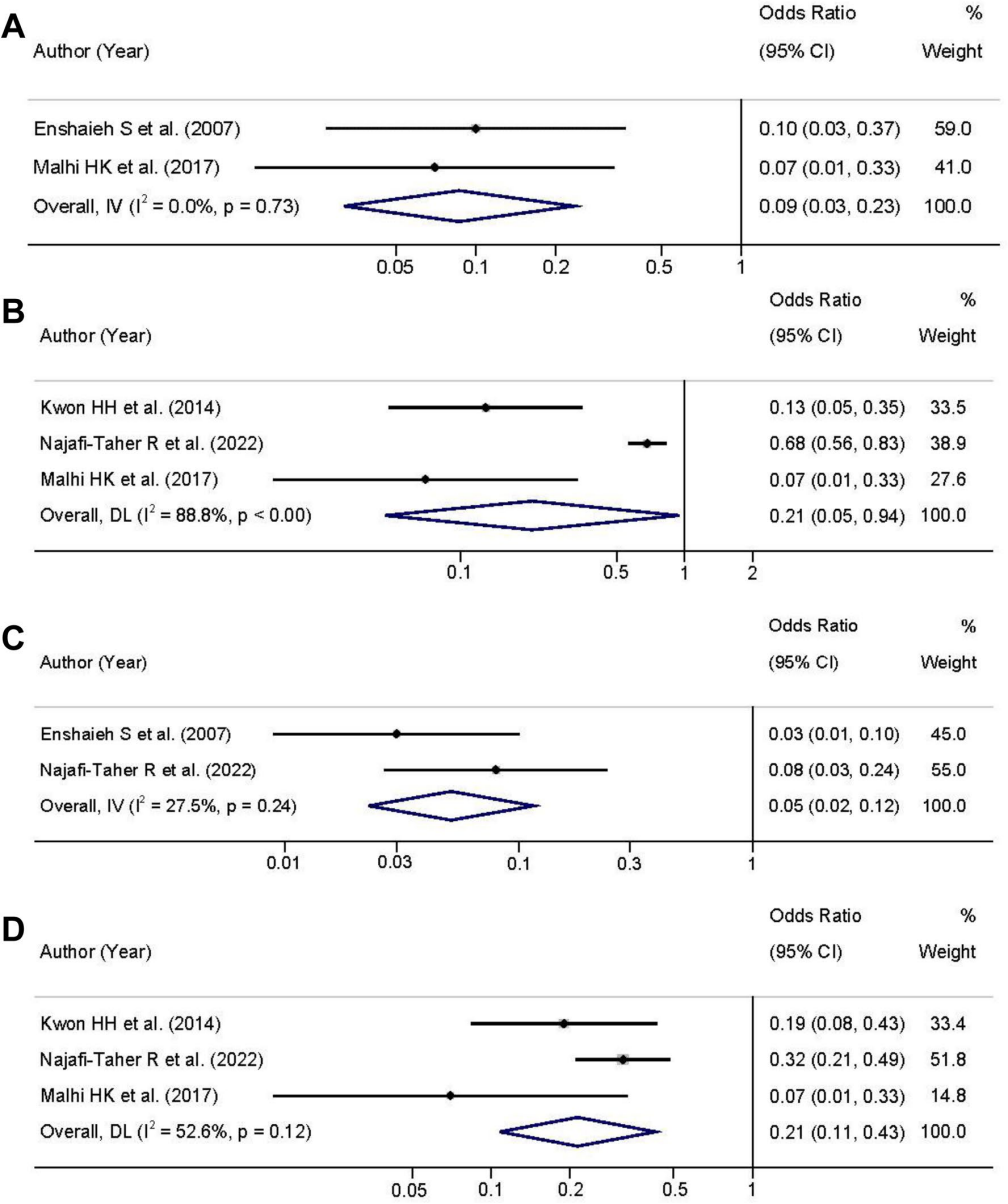
Forest plot. (A) Forestplot of mild itching associated with Tea Tree Oil (TTO) in acne treatment. (B) Forestplot of mild dryness associated with TTO in acne treatment. (C) Forestplot of mild burning sensation and irritation associated with TTO in acne treatment. (D) Forestplot of mild erythema and scaling associated with TTO in acne treatment.

The safety profile of TTO in acne treatment was comprehensively evaluated by analyzing several adverse events, including mild itching, dryness, burning sensation and irritation, and erythema and scaling. For mild itching, an exploratory meta‐analysis (Figure 3A), which included data from one RCT and one single‐arm study, yielded pOR of 0.09 (95% CI: 0.03–0.23). This suggests a lower incidence of mild itching associated with TTO. The statistical heterogeneity was low (*I* ^2^ = 0.0%, *p* = 0.73). The pooled effect was statistically significant (*z* = 4.80, *p* < 0.01), which is consistent with the observed trend of lower reported itching with TTO use. For mild dryness, an exploratory synthesis of three studies (Figure 3B) showed a combined OR of 0.21 (95% CI: 0.05–0.94), derived from a random‐effects model, suggesting a lower risk associated with TTO treatment. Despite substantial heterogeneity (*I* ^2^ = 88.8%, *p* < 0.01), which was likely attributable to variations in study populations, treatment regimens, or assessment methods, the overall effect size remained statistically significant (*z* = 2.04, *p* = 0.04). No significant publication bias was detected by Egger's test (bias coefficient = −3.63, *p* = 0.07 > 0.05) (Table S2), despite some asymmetry in the funnel plot (Figure S3), further supporting the robustness of these findings. Due to the considerable heterogeneity and limited number of studies, subgroup or meta‐regression analyses were not conducted. This quantitative synthesis indicates a potential benefit of TTO in alleviating mild dryness, though the estimate should be interpreted with caution due to heterogeneity.

Regarding mild burning sensations and irritation, an exploratory synthesis study (Figure 3C) revealed a combined OR of 0.05 (95% CI: 0.02–0.12), indicating a markedly lower risk of these adverse events with TTO treatment. The heterogeneity test indicated low between‐study variability (*I* ^2^ = 27.5%, *p* = 0.24), supporting the consistency of the findings. The effect size was statistically significant (*z* = 7.14, *p* < 0.01). These results are consistent with a reduction in the incidence of burning sensations and irritation associated with TTO use during acne treatment. For mild erythema and scaling, an exploratory synthesis of three studies (Figure 3D) showed a combined OR of 0.21 (95% CI: 0.11–0.43), derived from a random‐effects model, suggesting a reduction associated with TTO treatment. Moderate heterogeneity was observed (*I* ^2^ = 52.6%, *p* = 0.12), potentially due to differences in study populations, treatment protocols, or assessment methods. The statistical test (*z* = 4.40, *p* < 0.01) indicated a significant effect. The pooled estimate supports a potential therapeutic effect of TTO on these skin reactions.

**FIGURE 4:**
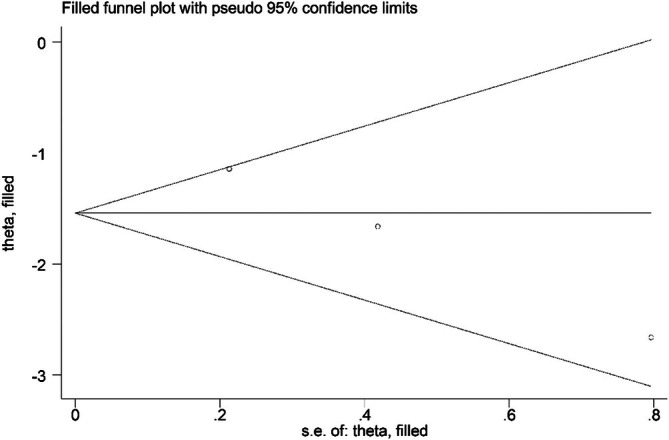
Filled funnel plot of mild erythema and scaling of Tea Tree Oil (TTO) on acne.

Asymmetry in the funnel plot (Figure S4), supported by a significant Egger's test (bias coefficient = −2.59, *p* < 0.01; Table S3), indicated the potential for publication bias. However, the trim‐and‐fill procedure (Figure 4), which iteratively imputes theoretically “missing” studies to achieve funnel plot symmetry, did not identify or add any studies in this case. This suggests that while statistical asymmetry exists, its magnitude and direction are not sufficient to materially alter the pooled effect estimate. Consequently, the adjusted pOR remained unchanged at 0.21 (95% CI: 0.11–0.43). Together, these analyses indicate that the observed beneficial effect of TTO in reducing erythema and scaling is robust, despite statistical indications of potential bias.

## 4 Discussion

To our knowledge, this is one of the earliest meta‐analyses to systematically evaluate the efficacy and safety of TTO in acne treatment. By synthesizing data from seven studies involving 445 participants, our analysis suggests that TTO is associated with a reduction in acne lesion counts and a lower ASI compared to control conditions. These findings indicate that TTO may serve as a topical option for patients who are intolerant to or prefer alternatives to conventional acne therapies. Across included studies, adverse events were predominantly mild and transient, with the most common being skin itching, dryness, erythema, and desquamation, with no serious adverse events reported.

Our findings are broadly consistent with previous reviews and narrative syntheses, supporting TTO's role in acne management (Nascimento et al. 2023). Earlier studies have reported reductions in inflammatory lesion counts and overall acne severity with TTO use (Bagherani and Smoller 2015), and some trials suggest comparable efficacy to benzoyl peroxide or adapalene, although with a slower onset of action (Salem et al. 2024). However, such comparisons remain preliminary, given the limited number of head‐to‐head trials and variability in formulations, concentrations, and outcome measures (Cao et al. 2015; Pezantes‐Orellana et al. 2025). Several recent reviews have similarly emphasized the scarcity of well‐powered, acne‐specific randomized controlled trials and the need for more rigorous clinical evaluation (Malhi et al. 2017; Nascimento et al. 2023).

The safety profile of TTO appears to be concentration dependent. Across studies, adverse events were generally mild and self‐limiting; however, the observed tolerability varied substantially (Bekhof et al. 2023; Crawford et al. 2004). Although pooled analyses suggested a lower incidence of certain local adverse events in TTO groups, these estimates were derived from heterogeneous studies and should be interpreted cautiously, particularly given indications of publication bias. Importantly, higher concentrations and inappropriate application have been associated with skin irritation and allergic contact dermatitis (Hammer et al. 2006). The substantial heterogeneity observed (e.g., *I* ^2^ = 88.8% for dryness) likely reflects wide variation in TTO concentration (5%–50%), formulation, treatment duration (4–12 weeks), and baseline acne severity (Malhi et al. 2017). These findings emphasize the need for standardized formulations and dosing regimens in future trials to enable reliable safety assessment.

The observed clinical effects are biologically plausible. Acne pathogenesis involves *C. acnes* proliferation, inflammation, follicular hyperkeratinization, and excess sebum production (Oliveira et al. 2025; Xu et al. 2025). TTO, particularly its major active component, terpinen‐4‐ol, exhibits broad‐spectrum antibacterial activity through disruption of bacterial membranes (Sharma et al. 2023) and anti‐inflammatory effects via suppression of proinflammatory cytokines such as TNF‐α and IL‐1β (Bagherani and Smoller 2015; Nascimento et al. 2023). Emerging evidence also suggests a potential seboregulatory effect (Hammer 2015). Together, these mechanisms provide a coherent rationale for the clinical associations observed in this analysis.

Several limitations of the existing evidence base should be acknowledged. Most trials enrolled small samples and focused predominantly on mild acne, limiting generalizability to moderate‐to‐severe disease (Kairey et al. 2023). There was considerable heterogeneity in study design, outcome assessment, and reporting of formulation details, complicating cross‐study comparisons (Carson et al. 2006; Nascimento et al. 2023). Geographic representation was also limited, with most studies conducted in Asian and Australian populations (Kairey et al. 2023; Nascimento et al. 2023). Short treatment durations (4–12 weeks) and the lack of long‐term safety data further constrain conclusions (Kairey et al. 2023; Nascimento et al. 2023). Additionally, blinding may have been compromised in some trials due to TTO's distinctive odor (Carson et al. 2006; Nascimento et al. 2023). Therefore, future studies should directly compare different TTO concentrations and vehicle bases to identify optimal formulations, enroll ethnically and geographically diverse populations to enhance generalizability, implement robust blinding methods, and include long‐term follow‐up to fully establish the efficacy and safety profile of TTO in acne management.

In summary, this systematic review and meta‐analysis suggests that TTO is associated with modest clinical benefits and acceptable short‐term tolerability in acne treatment, supporting its potential role as a topical alternative or adjunct. However, the evidence remains limited by heterogeneity and methodological constraints. Future research should prioritize well‐designed, adequately powered randomized trials with standardized formulations, robust blinding strategies, diverse populations, and longer‐term follow‐up to more definitively establish the therapeutic role and safety profile of TTO in acne management.

## Author Contributions

**Yixin Ye:** writing – original draft, data curation, writing – review and editing, visualization, formal analysis, validation, software, methodology, investigation. **Jun Jie Lim:** software, data curation, investigation, validation, formal analysis, visualization, writing – original draft, writing – review and editing, methodology, supervision. **Zongxun Huang:** data curation, software, writing – review and editing, investigation. **Fook Tim Chew:** conceptualization, resources, project administration, writing – review and editing, supervision, funding acquisition.

## Funding

Fook Tim Chew (F.T.C.) received grants from the National University of Singapore (N‐154‐000‐038‐001 (E‐154‐00‐0017‐01); C141‐000‐077‐001 (E‐141‐00‐0096‐01)), Singapore Ministry of Education Academic Research Fund (R‐154‐000‐191‐112; R‐154‐000‐404‐112; R‐154‐000‐553‐112; R‐154‐000‐565‐112; R‐154‐000‐630‐112; R‐154‐000‐A08‐592; R‐154‐000‐A27‐597; R‐154‐000‐A91‐592; R‐154‐000‐A95‐592; R‐154‐000‐B99‐114), Biomedical Research Council (BMRC) (Singapore) (BMRC/01/1/21/18/077; BMRC/04/1/21/19/315; BMRC/APG2013/108), Singapore Immunology Network (SIgN‐06‐006; SIgN‐08‐020), National Medical Research Council (NMRC) (Singapore) (NMRC/1150/2008; OFIRG20nov‐0033; MOH‐001636 (OFLCG23may‐0038, A‐8002641‐00‐00)), National Research Foundation (NRF) (Singapore) (NRF‐MP‐2020‐0004), Singapore Food Agency (SFA) (SFS_RND_SUFP_001_04; W22W3D0006; NRF‐SFSRND2SIH‐0001, SFS_RND_2_FS_0002), Singapore's Economic Development Board (EDB) (A‐8002576‐00‐00), and the Agency for Science Technology and Research (A*STAR) (Singapore) (H17/01/a0/008; and APG2013/108). This research is supported by the National Research Foundation Singapore under its Open Fund‐Large Collaborative Grant (MOH‐001636) (A‐8002641‐00‐00) and administered by the Singapore Ministry of Health's National Medical Research Council. The funding agencies had no role in the study design, data collection and analysis, decision to publish, or preparation of the manuscript.

## Conflicts of Interest

F.T.C. reports grants from the National University of Singapore, Singapore Ministry of Education Academic Research Fund, Singapore Immunology Network, National Medical Research Council (NMRC) (Singapore), Biomedical Research Council (BMRC) (Singapore), National Research Foundation (NRF) (Singapore), Singapore Food Agency (SFA), Singapore's Economic Development Board (EDB), and the Agency for Science Technology and Research (A*STAR) (Singapore), during the conduct of the study; and consulting fees from Sime Darby Technology Centre, First Resources Ltd., Genting Plantation, Olam International, Musim Mas, and Syngenta Crop Protection, outside the submitted work. The other authors declare no conflicts of interest. This research is supported by the National Research Foundation Singapore under its Open Fund‐Large Collaborative Grant (MOH‐001636) (A‐8002641‐00‐00) and administered by the Singapore Ministry of Health's National Medical Research Council. None of the authors has a conflicts of interest to declare.

## Supporting information


**Data S1:** ptr70344‐sup‐0001‐Supinfo1.docx.

**Figure S1:** (A) Risk of bias assessment. (B) Risk of bias assessment for individual studies.
**Figure S2:** Funnel plot of all included studies of the efficacy of TTO on acne treatment.
**Figure S3:** Funnel plot of all included studies of mild dryness.
**Figure S4:** Funnel plot of all included studies of erythema and scaling.
**Table S1:** Reason for exclusion of studies in the meta‐analysis.
**Table S2:** Egger's test of mild dryness of TTO on acne.
**Table S3:** Egger's test of mild erythema and scaling of TTO on acne.

## Data Availability

The data that support the findings of this study are available in the Supporting Information of this article.
