# Supplementary material for: Efficacy and Safety of Melaleuca alternifolia (Tea Tree) Oil for Acne—A Systematic Review and Meta‐Analysis
Source: Phytother Res. 2026 May 7;40(7):4444–53. doi: 10.1002/ptr.70344 (PMC13340978; doi:10.1002/ptr.70344)
Supplement: Supplementary file 1 — Data S1: ptr70344‐sup‐0001‐Supinfo1.docx. [file PTR-40-4444-s002.docx]

| **Section and Topic** | **Item #** | **Checklist item** | **Location where item is reported** |
| --- | --- | --- | --- |
| **TITLE** | | |  |
| Title | 1 | Identify the report as a systematic review. | The title is: "Efficacy and safety of *Melaleuca alternifolia* (tea tree) oil for acne — A systematic review and meta-analysis". |
| **ABSTRACT** | | |  |
| Abstract | 2 | See the PRISMA 2020 for Abstracts checklist. | The structured abstract includes: Background, Methods (search, inclusion criteria, synthesis methods), Results (number of studies and participants, pooled ORs), and Conclusion. |
| **INTRODUCTION** | | |  |
| Rationale | 3 | Describe the rationale for the review in the context of existing knowledge. | Stated in the Introduction (end of Section 1), highlighting acne burden, TTO's antimicrobial/anti‑inflammatory properties, and gaps in prior evidence. |
| Objectives | 4 | Provide an explicit statement of the objective(s) or question(s) the review addresses. | Explicitly stated at the end of Introduction (Section 1) and in the Abstract: to evaluate efficacy and safety of TTO for acne vulgaris. |
| **METHODS** | | |  |
| Eligibility criteria | 5 | Specify the inclusion and exclusion criteria for the review and how studies were grouped for the syntheses. | Detailed in Section 2.2 (Study Selection). Inclusion: human clinical trials, TTO for acne, reporting lesion counts/ASI. Exclusion: non‑original, non‑human, duplicate, insufficient data, non‑English. |
| Information sources | 6 | Specify all databases, registers, websites, organisations, reference lists and other sources searched or consulted to identify studies. Specify the date when each source was last searched or consulted. | Listed in Section 2.1: PubMed, Embase, Web of Science, Cochrane Library, Ovid. Search up to August 8, 2025. |
| Search strategy | 7 | Present the full search strategies for all databases, registers and websites, including any filters and limits used. | Full PubMed strategy provided in Table 1. Strategies for other databases adapted as described in Section 2.1. |
| Selection process | 8 | Specify the methods used to decide whether a study met the inclusion criteria of the review, including how many reviewers screened each record and each report retrieved, whether they worked independently, and if applicable, details of automation tools used in the process. | Two independent reviewers (YYX, JJL) screened titles/abstracts and full texts. Disagreements resolved by third reviewer (FTC). Described in Section 2.2. |
| Data collection process | 9 | Specify the methods used to collect data from reports, including how many reviewers collected data from each report, whether they worked independently, any processes for obtaining or confirming data from study investigators, and if applicable, details of automation tools used in the process. | Data extracted independently by two reviewers. Discrepancies resolved by consensus or third reviewer (FTC). Section 2.3. |
| Data items | 10a | List and define all outcomes for which data were sought. Specify whether all results that were compatible with each outcome domain in each study were sought (e.g. for all measures, time points, analyses), and if not, the methods used to decide which results to collect. | Primary outcomes: reduction in inflammatory acne lesions and Acne Severity Index (ASI). Secondary outcomes: adverse events (itching, dryness, erythema, irritation, burning). Section 2.3. |
|  | 10b | List and define all other variables for which data were sought (e.g. participant and intervention characteristics, funding sources). Describe any assumptions made about any missing or unclear information. | Extracted variables: study ID, design, intervention/comparator details, participant characteristics, funding source. Assumptions resolved by consensus. Section 2.3. |
| Study risk of bias assessment | 11 | Specify the methods used to assess risk of bias in the included studies, including details of the tool(s) used, how many reviewers assessed each study and whether they worked independently, and if applicable, details of automation tools used in the process. | Cochrane RoB 2 tool used. Two reviewers (YYX, ZXH) independently assessed each study. Disagreements resolved by third reviewer (FTC). Section 2.3. |
| Effect measures | 12 | Specify for each outcome the effect measure(s) (e.g. risk ratio, mean difference) used in the synthesis or presentation of results. | Odds Ratio (OR) with 95% CI used for all dichotomous outcomes (efficacy and safety). Section 2.4. |
| Synthesis methods | 13a | Describe the processes used to decide which studies were eligible for each synthesis (e.g. tabulating the study intervention characteristics and comparing against the planned groups for each synthesis (item #5)). | Studies meeting inclusion criteria and providing usable data for the binary outcome (≥50% reduction) were included in meta‑analysis. Sections 2.2, 2.4, 3.1. |
|  | 13b | Describe any methods required to prepare the data for presentation or synthesis, such as handling of missing summary statistics, or data conversions. | A post‑hoc binary outcome (≥50% reduction in acne lesions or ASI) defined to pool across studies with varied metrics. Section 2.4. |
|  | 13c | Describe any methods used to tabulate or visually display results of individual studies and syntheses. | Results presented in Table 2 (study characteristics), Figure 1 (flow diagram), Figures 2 & 3 (forest plots), Supplementary Figures (funnel plots). Section 2.4. |
|  | 13d | Describe any methods used to synthesize results and provide a rationale for the choice(s). If meta-analysis was performed, describe the model(s), method(s) to identify the presence and extent of statistical heterogeneity, and software package(s) used. | Fixed‑effect model used if I²≤50% and p>0.10; otherwise random‑effects. Heterogeneity assessed by Chi‑square and I². Software: Stata 14.0. Section 2.4. |
|  | 13e | Describe any methods used to explore possible causes of heterogeneity among study results (e.g. subgroup analysis, meta-regression). | Planned subgroup analyses (e.g., by TTO concentration) were not conducted due to the limited number of studies (n=7). Potential causes are discussed narratively in the Discussion (Sections 2.4, Discussion). |
|  | 13f | Describe any sensitivity analyses conducted to assess robustness of the synthesized results. | Two sensitivity analyses were planned and performed: (1) comparing fixed‑effect vs. random‑effects model results, and (2) assessing the impact of excluding studies judged to be at high risk of bias (Section 2.4). |
| Reporting bias assessment | 14 | Describe any methods used to assess risk of bias due to missing results in a synthesis (arising from reporting biases). | Publication bias assessed by Egger's test and funnel plots; trim‑and‑fill used to adjust. Sections 2.4 & 3.4. |
| Certainty assessment | 15 | Describe any methods used to assess certainty (or confidence) in the body of evidence for an outcome. | No formal GRADE assessment; certainty discussed narratively in Discussion. |
| **RESULTS** | | |  |
| Study selection | 16a | Describe the results of the search and selection process, from the number of records identified in the search to the number of studies included in the review, ideally using a flow diagram. | Detailed in Section 3.1 and Figure 1: 344 records identified, 7 studies in qualitative synthesis, 4 in quantitative synthesis (meta‑analysis). |
|  | 16b | Cite studies that might appear to meet the inclusion criteria, but which were excluded, and explain why they were excluded. | Excluded full‑text studies and reasons listed in Supplementary Table 1 (Section 3.1). |
| Study characteristics | 17 | Cite each included study and present its characteristics. | Characteristics of 7 included studies summarized in Table 2 (Section 3.1). |
| Risk of bias in studies | 18 | Present assessments of risk of bias for each included study. | RoB 2 assessments summarized in Section 3.2 and detailed in Supplementary Figure 1. |
| Results of individual studies | 19 | For all outcomes, present, for each study: (a) summary statistics for each group (where appropriate) and (b) an effect estimate and its precision (e.g. confidence/credible interval), ideally using structured tables or plots. | Efficacy: responder/non‑responder counts in forest plot (Figure 2). Safety: event counts in forest plots (Figures 3A‑D). |
| Results of syntheses | 20a | For each synthesis, briefly summarise the characteristics and risk of bias among contributing studies. | The studies contributing to the main efficacy synthesis (n=4) and safety syntheses are described in Sections 3.1 and 3.2. The overall risk of bias was judged as low for most domains, though some concerns were noted in allocation concealment and blinding (Section 3.2). |
|  | 20b | Present results of all statistical syntheses conducted. If meta-analysis was done, present for each the summary estimate and its precision (e.g. confidence/credible interval) and measures of statistical heterogeneity. If comparing groups, describe the direction of the effect. | Efficacy: pooled OR = 0.74 (95% CI: 0.63–0.86), I²=0% (Figure 2, Section 3.3). Safety: e.g., itching OR=0.09 (0.03–0.23), I²=0%; dryness OR=0.21 (0.05–0.94), I²=88.8% (Figure 3, Section 3.4). |
|  | 20c | Present results of all investigations of possible causes of heterogeneity among study results. | No formal investigations due to limited study numbers; stated in Section 3.4. |
|  | 20d | Present results of all sensitivity analyses conducted to assess the robustness of the synthesized results. | Sensitivity analyses: fixed‑effect vs. random‑effects gave consistent conclusions; excluding high‑bias studies did not alter primary efficacy estimate. Section 3.3. |
| Reporting biases | 21 | Present assessments of risk of bias due to missing results (arising from reporting biases) for each synthesis assessed. | Publication bias was assessed using Egger’s test and funnel plots. For the primary efficacy outcome, Egger‘s test was not significant (p=0.459). For the erythema/scaling outcome, trim-and-fill was performed; for dryness, Egger’s test was borderline (p=0.07). Details in Sections 3.3, 3.4, and Supplementary Figures 2-4. |
| Certainty of evidence | 22 | Present assessments of certainty (or confidence) in the body of evidence for each outcome assessed. | Certainty described narratively as "moderate" or "preliminary" in Abstract and Conclusion; limitations discussed in Discussion. |
| **DISCUSSION** | | |  |
| Discussion | 23a | Provide a general interpretation of the results in the context of other evidence. | Discussion interprets findings, compares with previous reviews, and provides biological rationale. |
|  | 23b | Discuss any limitations of the evidence included in the review. | Limitations of included studies (small samples, varied formulations, short duration, geographic concentration) discussed in Discussion. |
|  | 23c | Discuss any limitations of the review processes used. | Review limitations (post‑hoc outcome definition, inability to conduct subgroup analyses, language bias) discussed in Discussion. |
|  | 23d | Discuss implications of the results for practice, policy, and future research. | Implications for clinical practice and future research (standardization, larger trials, long‑term safety) provided in Discussion and Conclusion. |
| **OTHER INFORMATION** | | |  |
| Registration and protocol | 24a | Provide registration information for the review, including register name and registration number, or state that the review was not registered. | PROSPERO registration CRD420251127442, stated in Section 2.1. |
|  | 24b | Indicate where the review protocol can be accessed, or state that a protocol was not prepared. | The protocol is accessible at: <https://www.crd.york.ac.uk/prospero/display_record.php?ID=CRD420251127442> |
|  | 24c | Describe and explain any amendments to information provided at registration or in the protocol. | No amendments made; stated in Section 2.1. |
| Support | 25 | Describe sources of financial or non-financial support for the review, and the role of the funders or sponsors in the review. | Funding sources listed in Section 7 (Fundings); funders had no role in study design, data collection, analysis, or manuscript preparation. |
| Competing interests | 26 | Declare any competing interests of review authors. | Competing interests declared in Section 8. FTC reports grants and consulting fees; other authors declare none. |
| Availability of data, code and other materials | 27 | Report which of the following are publicly available and where they can be found: template data collection forms; data extracted from included studies; data used for all analyses; analytic code; any other materials used in the review. | All data generated or analyzed during this study are included in this published article and its supplementary information files. The full search strategy is provided in Table 1. No additional data or code are publicly available. (Data availability statement in manuscript, after Conflicts of interest section.) |

*From:*  Page MJ, McKenzie JE, Bossuyt PM, Boutron I, Hoffmann TC, Mulrow CD, et al. The PRISMA 2020 statement: an updated guideline for reporting systematic reviews. BMJ 2021;372:n71. doi: 10.1136/bmj.n71
