# Supplementary material for: Efficacy and Safety of Melaleuca alternifolia (Tea Tree) Oil for Acne—A Systematic Review and Meta‐Analysis
Source: Phytother Res. 2026 May 7;40(7):4444–53. doi: 10.1002/ptr.70344 (PMC13340978; doi:10.1002/ptr.70344)
Supplement: Supplementary file 2 — Figure S1: (A) Risk of bias assessment. (B) Risk of bias assessment for individual studies. Figure S2: Funnel plot of all included studies of the efficacy of TTO on acne treatment. Figure S3: Funnel plot of all included studies of mild dryness. Figure S4: Funnel plot of all included studies of erythema and scaling. Table S1: Reason for exclusion of studies in the meta‐analysis. Table S2: Egger's test of mild dryness of TTO on acne. Table S3: Egger's test of mild erythema and scaling of TTO on acne. [file PTR-40-4444-s001.docx]

**Supplementary Materials**

**Efficacy and safety of Melaleuca alternifolia (tea tree) oil for acne—A systematic review and meta-analysis**

YixinYe^1,2^, Jun Jie Lim^2^, Zongxun Huang^2^, Fook Tim Chew^2^*

^1^School of Public Health and Management, Guangxi University of Chinese Medicine, Nanning, China;

^2^Department of Biological Sciences, National University of Singapore, Singapore.

*Correspondence to: Fook Tim Chew, Department of Biological Sciences, Faculty of Science, National University of Singapore, 14 Science Drive 4, Block S2, Level 5, Singapore 117543. Email: dbscft@nus.edu.sg


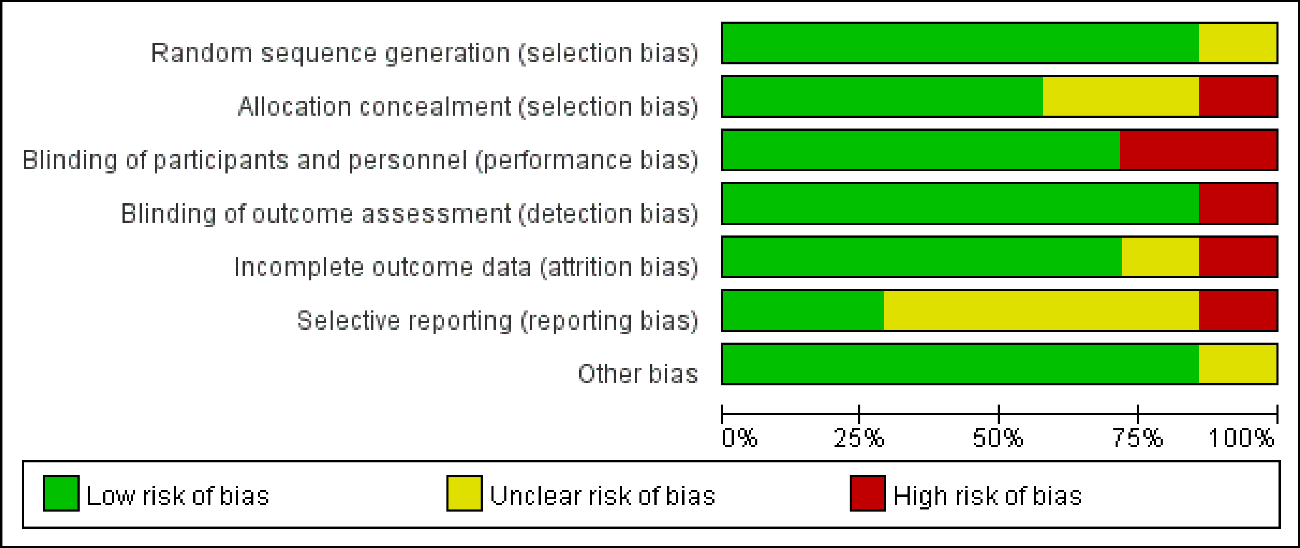


**Supplementary Figure 1A risk of bias assessment.**


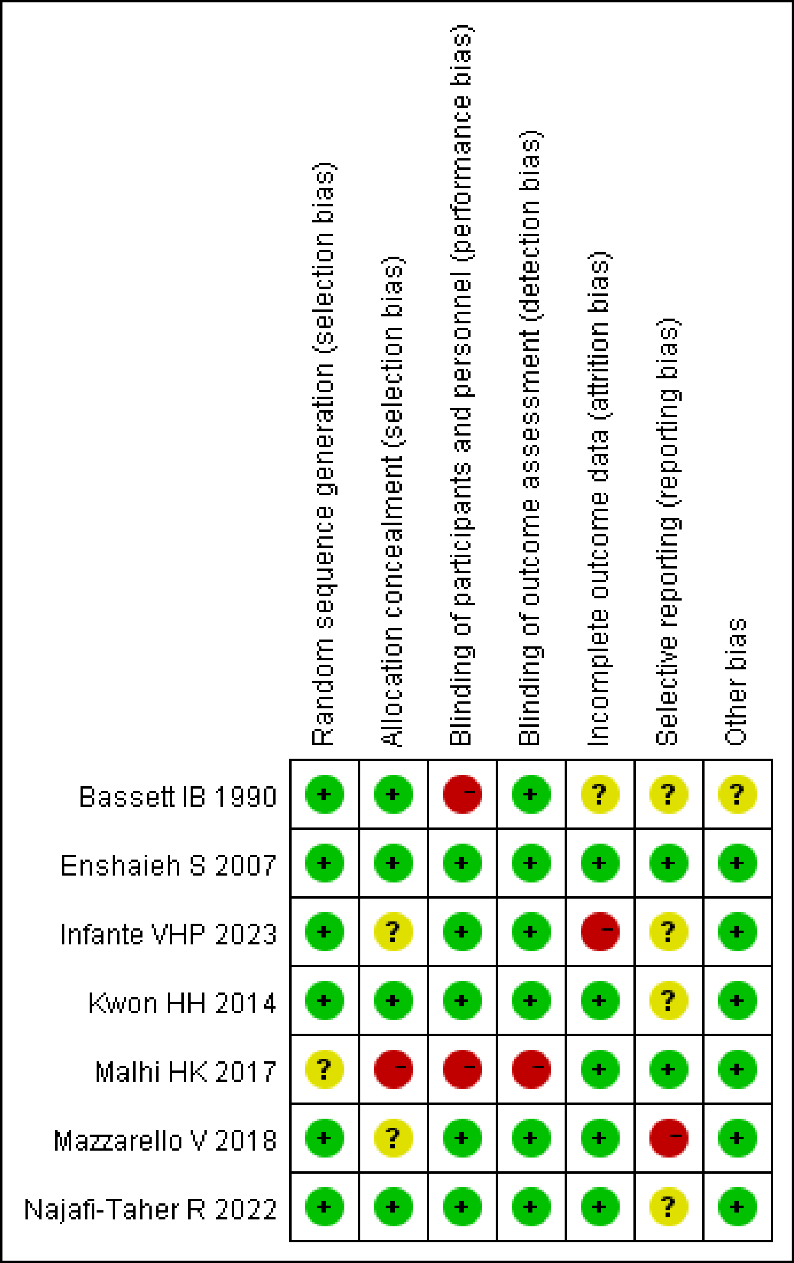


**Supplementary Figure 1B Risk of bias assessment for Individual Studies.**

**Note for Supplementary Figures 2-4:**

Due to the limited number of included studies (n < 10), the funnel plots and Egger’s tests have low statistical power, and the results on publication bias should be interpreted with caution.


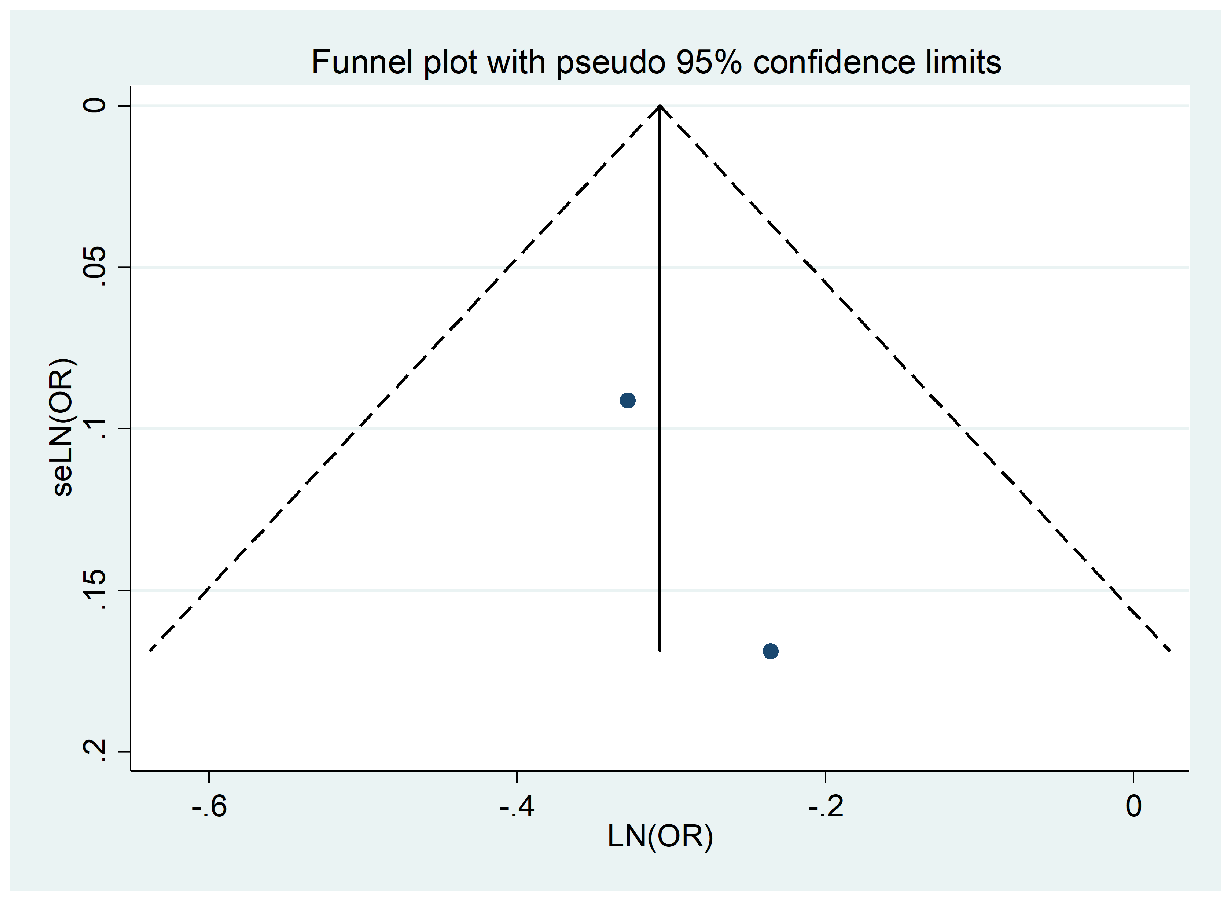


**Supplementary Figure 2 Funnel plot of all included studies of the Efficacy of TTO on Acne Treatment.**


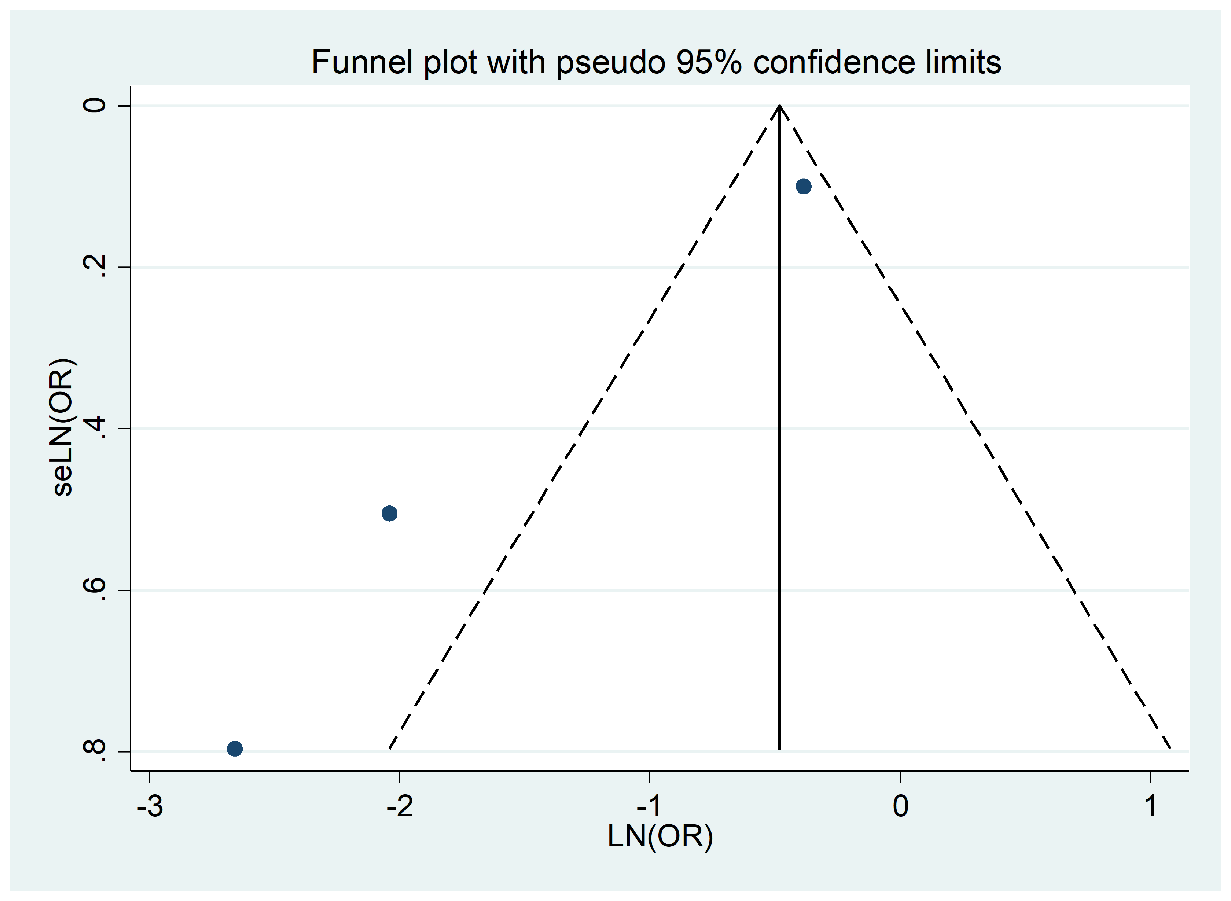


**Supplementary Figure 3 Funnel plot of all included studies of mild dryness.**


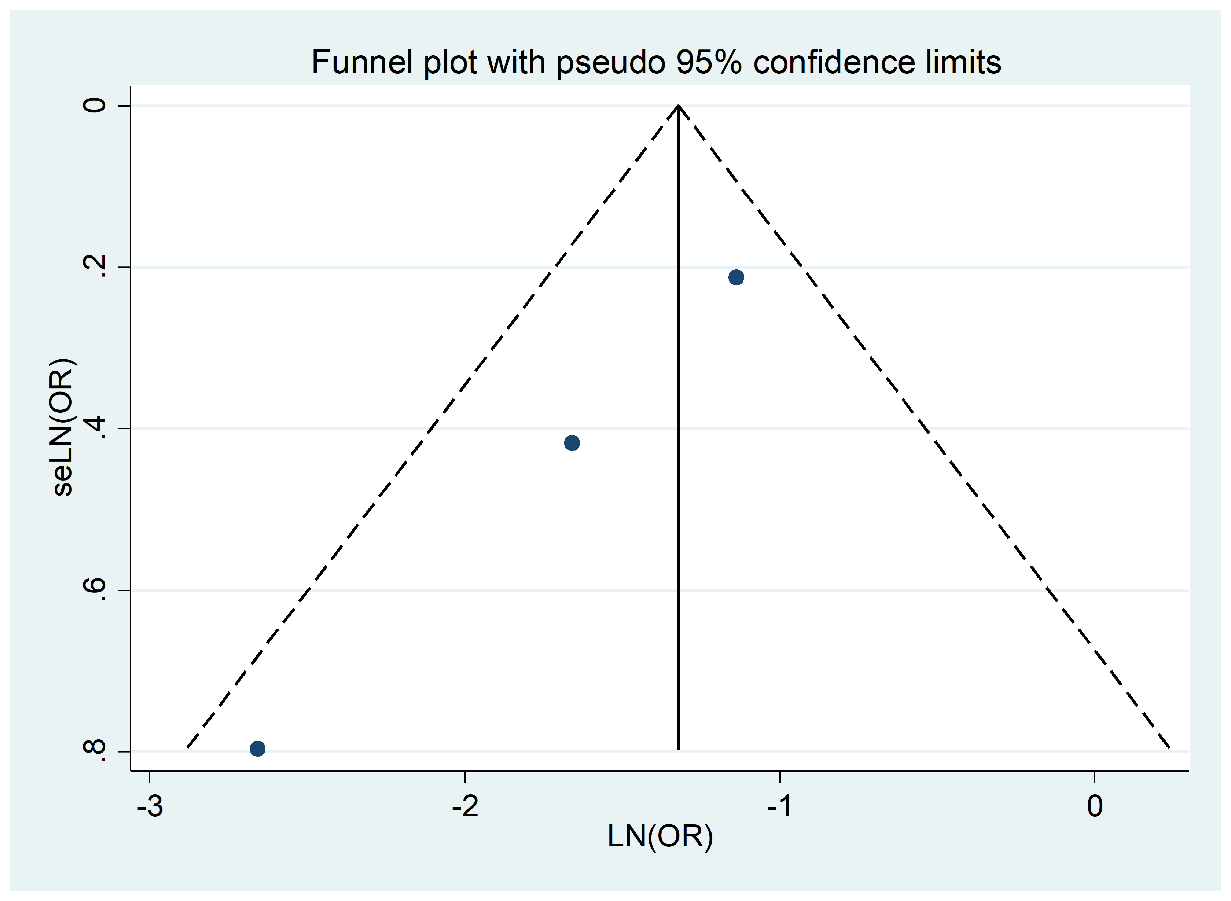


**Supplementary Figure 4 Funnel plot of all included studies of erythema and scaling.**

**Supplementary Table1 Reason for exclusion of studies in the meta-analysis.**

| **Study** | **Reason for Exclusion** |
| --- | --- |
| Mazzarello V 2018 | Did not provide sufficient data on the total acne lesion count and Acne Severity Index (ASI) for meta-analysis. |
| Infante VHP 2023 | Used a different formulation of tea tree oil (nanoemulsion with 2% concentration), which was not comparable to the other studies in terms of treatment protocol and outcome measures. |
| Malhi HK 2017 | The study design was unique, with a very small sample size (14 treatment participants) and the absence of a formal control group, making it unsuitable for inclusion in the meta-analysis. |

**Supplementary Table 2 Egger's test of Mild Dryness of TTO on Acne.**

| **Egger's test** |  |  |  |  |  | |
| --- | --- | --- | --- | --- | --- | --- |
| Std_Eff | Coef. | Std. Err. | *t* | *P*>\|*t*\| | 95% Conf. Interval | |
| Slope | -0.02 | 0.07 | -0.34 | 0.79 | -0.92 | 0 .87 |
| Bias | -3.63 | 0.42 | -8.73 | 0.07 | -8.93 | 0.66 |

**Supplementary Table 3 Egger's test of Mild Erythema and Scaling of TTO on Acne.**

| **Egger's test** |  |  |  |  |  | |
| --- | --- | --- | --- | --- | --- | --- |
| Std_Eff | Coef. | Std. Err. | *t* | *P*>\|*t*\| | 95% Conf. Interval | |
| Slope | -0.59 | 0.01 | -57.62 | 0.01 | -0.72 | -0.46 |
| Bias | -2.59 | 0.03 | -80.90 | 0.008 | -2.99 | -2.18 |
